# Replication-coupled hemimethylation in *Escherichia coli* K-12: mechanisms, dynamics, and emerging opportunities for direct observation

**DOI:** 10.3389/fmicb.2026.1882318

**Published:** 2026-06-23

**Authors:** Chang Hou, Runsheng Li

**Affiliations:** Department of Infectious Diseases and Public Health, Jockey Club College of Veterinary Medicine and Life Sciences, City University of Hong Kong, Kowloon, Hong Kong SAR, China

**Keywords:** Dam methyltransferase, DNA methylation, duplex sequencing, *Escherichia coli* K-12, hemimethylation, mismatch repair, nanopore sequencing, phase variation

## Abstract

Bacterial DNA methylation has often been viewed as a relatively stable, motif-specific modification involved in restriction-modification systems, genome marking, and selected regulatory processes. However, in *Escherichia coli* K-12, several classical Dam-dependent mechanisms show that the biological significance of methylation can also arise from transient hemimethylated states generated immediately after DNA replication. Because these states are short-lived and strand-paired, they remained difficult to directly characterize with classical genetic, biochemical, and restriction-based assays. In this review, we characterize replication-coupled hemimethylation as a short-lived, strand-asymmetric DNA configuration that is interpreted by cellular machineries. We focus on three well-characterized systems in *E. coli* K-12: *oriC*/SeqA-dependent replication initiation control, methyl-directed mismatch repair, and Dam-dependent phase variation at loci such as *agn43* and *pap*. These examples demonstrate that hemimethylation can function as a timing signal, a strand-discrimination cue, or a post-replicative competition window for regulatory protein binding. We also discuss why classical assays largely inferred hemimethylation rather than directly observing strand-paired methylation states. Finally, we consider how single-molecule and duplex-aware sequencing technologies, including SMRT and nanopore-based approaches, now provide new opportunities to revisit bacterial hemimethylation at higher molecular resolution. These advances may help clarify unresolved questions in Dam-dependent systems and reveal whether replication-coupled hemimethylation extends beyond the classical mechanisms described in *E. coli* K-12.

## 1 Introduction

DNA methylation is among the most extensively studied epigenetic modifications (Sanchez-Romero and Casadesus, 2020). In eukaryotes, DNA methylation has long been associated with transcriptional silencing, chromatin organization, and developmental regulation, and its roles in gene regulation have therefore been investigated in considerable detail (Lawrence et al., 2016). By contrast, although bacterial DNA methylation was discovered early and is now known to be widespread across prokaryotes, it was historically interpreted largely through restriction–modification systems, motif-specific genome marking (Blow et al., 2016; Lobner-Olesen et al., 2005). However, in certain bacterial systems, the functional significance of DNA methylation arises not only from stably maintained fully methylated DNA, but also from transient methylation-state transitions generated following DNA replication. This distinction is particularly evident in orphan methyltransferase systems, in which DNA methylation is not coupled to cognate restriction endonucleases and lacks a eukaryote-like active demethylation framework (Sanchez-Romero and Casadesus, 2020). Under these conditions, DNA replication itself becomes the major driver of methylation-state transitions.

In *Escherichia coli* K-12, this replication-coupled methylation logic has been dissected most clearly. Following semiconservative DNA replication, the parental strand retains methylation whereas the newly synthesized strand remains transiently unmethylated, thereby generating a transient and strand-asymmetric hemimethylated state (Marinus and Løbner-Olesen, 2014). Rather than representing a simple biochemical intermediate, this replication-generated state functions as an informational DNA configuration that can be selectively interpreted by proteins able to discriminate methylated from hemimethylated binding sites (Sanchez-Romero and Casadesus, 2020). Classical studies in *E. coli* have demonstrated that several major chromosome-associated processes depend on this transient hemimethylated state, including chromosome replication, mismatch repair, and methylation-dependent phase variation systems (Blyn et al., 1989; Lobner-Olesen et al., 2005). In these mechanisms, hemimethylation is not merely a byproduct of replication, but a post-replicative signal read by chromosome-associated machineries. Despite decades of study, evidence for these mechanisms has relied largely on indirect approaches, including genetic perturbations, restriction-based assays, remethylation kinetics, footprinting, and reporter readouts. Thus, classical work established hemimethylation as a functional component of Dam-dependent regulation before its transient, strand-paired molecular form could be directly resolved.

Recent advances in single-molecule methylome sequencing, including SMRT and nanopore sequencing, have transformed bacterial methylation analysis by enabling high-throughput, genome-wide detection of methylated bases from native DNA (Beaulaurier et al., 2015; Jain et al., 2016). More recent duplex-aware strategies now provide a conceptual and technical route toward strand-paired observation of hemimethylated states (Halliwell et al., 2025; Liu et al., 2025). However, direct genome-wide analysis of transient bacterial 6mA hemimethylation remains an emerging application. Nanopore sequencing is especially relevant to this question because ONT reads preserve long native DNA molecules and, in duplex-aware workflows, can begin to connect modified-base calls to complementary strands from the same original duplex.

In this review, we deliberately center *E. coli* K-12 as a model system to examine the formation and functions of replication-coupled methylation states, rather than attempting a broad survey of all bacterial methylation systems. We emphasize how hemimethylation acts as a transient yet interpretable DNA state in key cellular processes, and how modern sequencing technologies are beginning to permit direct observation.

## 2 The methylation landscape of *Escherichia coli* K-12

In bacteria, DNA methylation occurs predominantly in three chemical forms: N6-methyladenine (6mA), 5-methylcytosine (5mC), and N4-methylcytosine (4mC), which are generated by DNA methyltransferases that transfer a methyl group from S-adenosyl-methionine to DNA (Jurkowska and Jeltsch, 2022). Among these modifications, 6mA represents the predominant modification in many bacterial methylomes, whereas 5mC and 4mC show more lineage-specific distributions (Blow et al., 2016).

Bacterial DNA methyltransferases can be broadly divided into two functional categories. One category consists of methyltransferases associated with restriction–modification (RM) systems, in which DNA methylation functions in self versus non-self discrimination and is coupled to cognate restriction endonucleases as part of host defense mechanisms. The other category comprises orphan methyltransferases, which lack associated restriction enzymes and include systems involved in chromosome-associated processes such as DNA replication, repair, and regulation (Blow et al., 2016; Murray, 2002).

In *E. coli* K-12, the detectable methylation landscape is dominated by Dam- and Dcm-dependent methylation at the abundant GATC and CCWGG motifs. The major DNA methyltransferase systems identified in *E. coli* K-12 are summarized in Table 1. Dam methylates adenine residues within 5′-GATC-3′ motifs and is responsible for the vast majority of N6-methyladenine (6mA) modifications in the genome (Marinus and Løbner-Olesen, 2014; Marinus and Morris, 1973). Because Dam modifies a short, abundant motif and its target sites are rapidly converted into hemimethylated dyads after replication, Dam provides the clearest system in *E. coli* K-12 for linking methylation-state dynamics to chromosome function. Classical studies have established that Dam-mediated methylation participates in multiple chromosome-associated processes, including *oriC* replication initiation control (Campbell and Kleckner, 1990; Slater et al., 1995), methyl-directed mismatch repair (Lahue et al., 1987; Lu et al., 1983), and Dam-dependent phase variation at loci such as *agn43* (Correnti et al., 2002; Haagmans and van der Woude, 2000).

**Table 1:** Major DNA methyltransferase systems identified in *E. coli* K-12.

Enzyme/System	Recognition motif	Major modification	Major associated functions
Dam	5′-GATC-3′	N6-methyladenine (6mA)	Chromosome replication initiation timing; methyl-directed mismatch repair; phase-variable transcription regulation
Dcm	5′-CCWGG-3′	5-methylcytosine (5mC)	Very-short-patch repair; stationary phase-associated transcriptional effects
EcoKI (HsdR, HsdM, HsdS)	5′-AAC(N₆)GTGC-3′	N6-methyladenine (6mA)	Host defense; self/non-self DNA discrimination
YhdJ	5′-ATGCAT-3′	N6-methyladenine (6mA)	Low or condition-dependent methylation activity; function incompletely characterized

Dcm methylates cytosine residues within 5′-CC (A/T)GG-3′(5′-CCWGG-3′) motifs, thereby generating 5-methylcytosine (5mC) (Marinus and Løbner-Olesen, 2014; Marinus and Morris, 1973). Current evidence primarily links Dcm to cytosine methylation-associated genome maintenance functions. The best-characterized example is its association with the very-short-patch repair pathway, which corrects T:G mismatches generated by spontaneous deamination of 5mC and thereby reduces transition mutations at CCWGG sites (Lieb and Bhagwat, 1996). More recent studies have further suggested a potential role for Dcm in growth phase-associated transcriptional regulation (Kahramanoglou et al., 2012).

In addition to orphan methyltransferase systems, *E. coli* K-12 also encodes the Type I restriction–modification (RM) system EcoKI, which is specified by the *hsdR*, *hsdM*, and *hsdS* genes (Murray, 2002; Roer et al., 2015). Type I RM systems typically consist of a restriction subunit, a methyltransferase subunit, and a sequence-specificity subunit, and function through sequence-specific DNA methylation to distinguish self from non-self DNA, thereby restricting the entry and propagation of foreign genetic material (Murray, 2002). EcoKI recognizes a bipartite DNA sequence, classically defined as 5′-AAC (N₆)GTGC-3′, and generates N6-methyladenine modifications at adenine residues located at defined positions (Roer et al., 2015). Because EcoKI recognition sequences are relatively long and far less frequent than Dam GATC or Dcm CCWGG motifs, EcoKI contributes a more restricted methylation pattern rather than a dense genome-wide modification landscape (Kelleher and Raleigh, 1991; Marinus and Løbner-Olesen, 2014). EcoKI restriction targets unmethylated recognition sites, although this activity can be abolished in laboratory derivatives carrying *hsdR* or *hsdS* mutations (Kelleher and Raleigh, 1991).

In addition, the *E. coli* genome encodes several predicted or condition-dependent methyltransferases, including YhdJ. YhdJ has been characterized as a nonessential orphan adenine methyltransferase that can methylate the NsiI-related sequence 5′-ATGCAT-3′ when overexpressed or assayed *in vitro*, but YhdJ-dependent methylation is below the limit of detection under standard laboratory K-12 conditions (Broadbent et al., 2007; Marinus and Løbner-Olesen, 2014).

**Figure 1:**
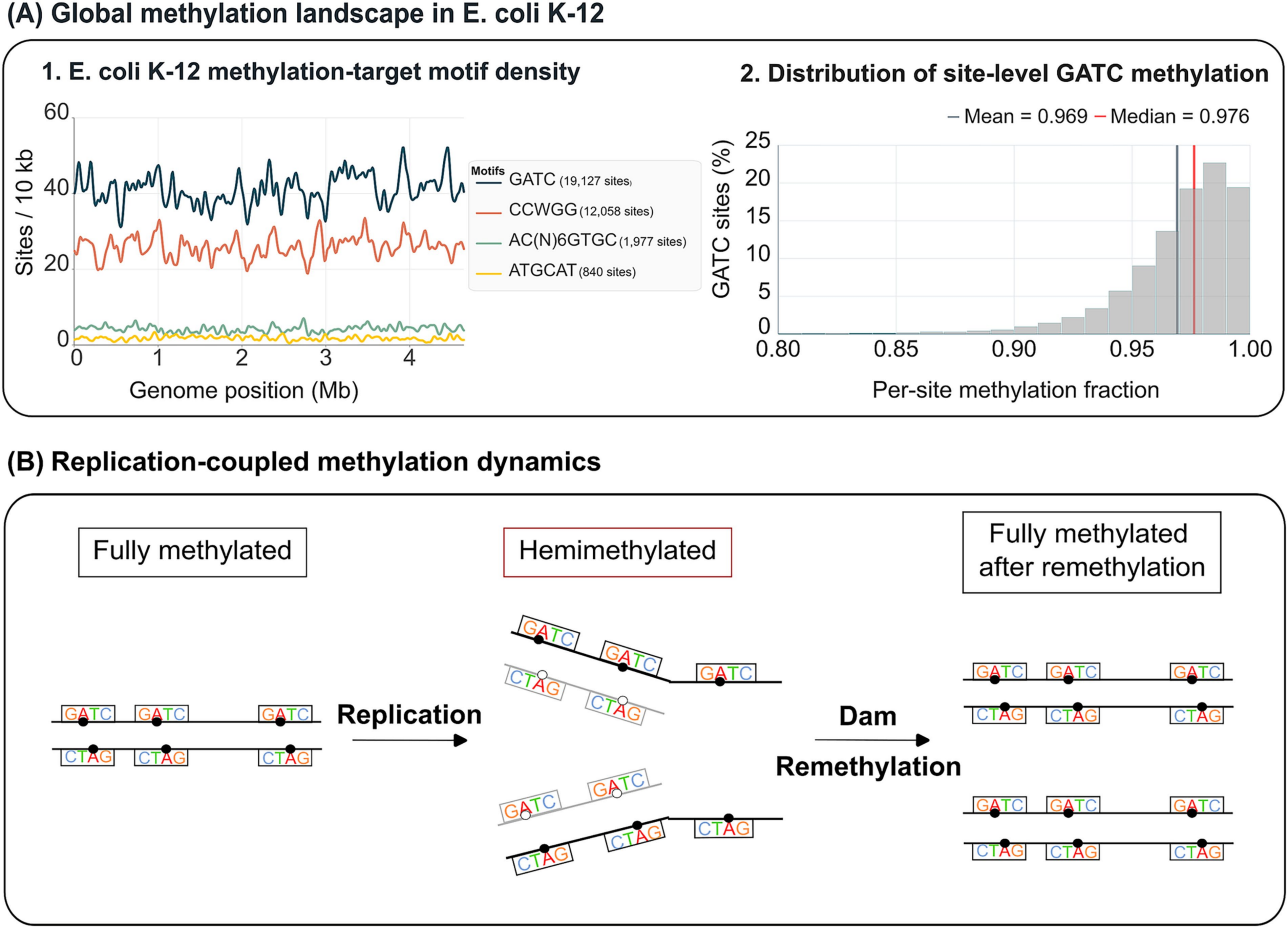
Global methylation landscape and replication-coupled methylation dynamics in *E. coli* K-12. **(A)** Overview of the motif-driven methylation landscape in *E. coli* K-12. **(A1)** Genome-wide densities of major methylation-target motifs across the chromosome. GATC motifs targeted by Dam and CCWGG motifs targeted by Dcm are highly abundant relative to motifs associated with EcoKI, or YhdJ. **(A2)** Distribution of site-level GATC methylation fractions, showing that most GATC sites are maintained in a near-fully methylated state under standard growth conditions. **(B)** Schematic of replication-coupled methylation-state transitions. Fully methylated GATC sites become transiently hemimethylated following semiconservative DNA replication because parental strands retain pre-existing methylation marks whereas newly synthesized strands initially lack methylation. Subsequent Dam-mediated remethylation restores the fully methylated state. Black circles indicate methylated adenines within GATC/CTAG dyads, and open circles indicate unmethylated target adenines on newly synthesized strands. This transient hemimethylated intermediate underlies multiple Dam-dependent cellular processes discussed in this Review. Motif-site counts were calculated from an MG1655-derived chromosome sequence deposited under GEO accession GSE206608 (Tang and Brynildsen, 2023). GATC methylation fraction data were derived from SMRT methylome datasets reported by Cohen et al. (2016).

Taken together, although multiple DNA methyltransferase systems are present in *E. coli* K-12, the overall methylation landscape is dominated by the high-occupancy methylation patterns generated by the orphan methyltransferases Dam and Dcm at GATC and CCWGG motifs (Figure 1A). Among these systems, the functional connections between Dam-mediated methylation and chromosome-associated processes are the most extensively characterized. Importantly, although these mechanisms were initially described primarily in terms of Dam methylation or GATC methylation, subsequent studies demonstrated that the molecular entities interpreted by regulatory proteins and repair machineries are often the transient hemimethylated states generated immediately following DNA replication (Campbell and Kleckner, 1990; Correnti et al., 2002; Lahue et al., 1987).

By contrast, Dcm is clearly functional in *E. coli* K-12, most notably through its association with very-short-patch repair and, more recently, with growth phase-associated transcriptional effects (Kahramanoglou et al., 2012). However, classical mechanisms in which replication-generated Dcm hemimethylated intermediates are explicitly read or maintained, comparable to those established for Dam-associated systems, have not yet been clearly identified. Accordingly, the following sections will focus primarily on replication-coupled hemimethylated states and their formation and functions within Dam-associated regulatory systems.

## 3 Replication-generated hemimethylation as a transient DNA state

In *E. coli* K-12, DNA methylation does not exist solely as a single static state, but instead continuously transitions through different methylation configurations during DNA replication. Because Dam and Dcm target motifs are maintained in a near-fully methylated state across most of the genome, fully methylated DNA can be considered the default configuration of the K-12 methylation landscape (Figure 1A). Following semiconservative DNA replication, the parental strand retains its original methylation marks whereas the newly synthesized strand remains transiently unmethylated immediately after synthesis, converting fully methylated sites into hemimethylated states (Herman and Modrich, 1982; Urig et al., 2002) (Figure 1B). DNA methyltransferases subsequently remethylate the nascent strand, restoring symmetric methylation at most target sites. In *E. coli* K-12, this post-replicative GATC hemimethylation window is usually short-lived because Dam is synthesized throughout growth and follows the replication fork within a short genomic distance, estimated to be less than 10 kbp (Boye et al., 1992; Campbell and Kleckner, 1988). Whereas most isolated GATC sites are rapidly remethylated following fork passage, clustered hemimethylated GATC regions can display slower remethylation kinetics. This delay is thought to arise when DNA-bound proteins, such as SeqA at clustered GATC regions including oriC and the *dnaA* promoter, preferentially bind hemimethylated sites and limit Dam access, thereby stabilizing transient hemimethylated states (Casadesus and Low, 2006; Low and Casadesus, 2008). When such protection persists across replication cycles, hemimethylated intermediates can give rise to locally unmethylated target motifs (Casadesus and Low, 2013; Sanchez-Romero et al., 2015). In this sense, unmethylated states in orphan methyltransferase systems are usually locus-specific products of methylation protection rather than genome-wide default states (Sanchez-Romero and Casadesus, 2020).

Together, this section establishes hemimethylation as a replication-generated state rather than a rare methylation defect. In *E. coli* K-12, fully methylated motifs are transiently converted into strand-asymmetric dyads after fork passage; most are rapidly remethylated, whereas selected loci can remain protected long enough to be interpreted by chromosome-associated proteins. This framework sets up the following examples, in which the same transient state is read as a timing signal, a strand-discrimination cue, or a regulatory competition window.

## 4 Biological interpretation of replication-coupled hemimethylation

The key question, therefore, is how cells read this short-lived methylation asymmetry. In *E. coli* K-12, hemimethylated DNA is not interpreted by a single universal reader, but by distinct proteins and repair machineries whose responses depend on local DNA context and biological function. The following sections examine three systems as representative examples of how replication-generated hemimethylation is interpreted as a timing signal at *oriC*, a strand-identity signal in mismatch repair, or a post-replicative competition window in phase-variable gene regulation.

### 4.1 Dam-dependent replication initiation control through hemimethylation

In *E. coli*, chromosomal replication initiates from a single defined origin, *oriC*. During rapid growth, overlapping rounds of replication can generate multiple *oriC* copies within the same cell, yet initiation remains tightly coordinated and each newly replicated origin is normally prevented from immediately firing again (Skarstad et al., 2000; Slater et al., 1995). Replication control therefore requires not only efficient origin activation, but also a post-initiation refractory period that prevents newly replicated origins from immediately reinitiating (Boye and Lobner-Olesen, 1990; Campbell and Kleckner, 1990). This control is closely linked to the methylation state of *oriC*. The *E. coli* chromosomal origin contains 11 GATC sites, which are maintained in a fully methylated configuration before initiation (Oka et al., 1984). Early studies using *oriC*-containing minichromosomes showed that these plasmids could not be stably maintained in *dam* mutants, establishing that Dam-mediated methylation is required for efficient *oriC* function (Messer et al., 1985). These studies also proposed that the time required for post-replicative remethylation of *oriC* could contribute to the minimal interval between successive initiation events, thereby linking replication timing to methylation-state transitions at the origin (Messer et al., 1985).

Dam methylation also affects the replication initiator gene *dnaA*. In *dam* mutants, *dnaA* transcription is substantially reduced, primarily through decreased activity of the proximal *dnaA2* promoter (Braun et al., 1985). Because the *dnaA* promoter region is also enriched in GATC sites, these findings suggested that Dam methylation contributes to replication initiation control through coordinated effects on both *oriC* activity and *dnaA* availability (Campbell and Kleckner, 1990). Consistent with this interpretation, initiation timing in *dam* mutants becomes poorly synchronized rather than tightly coordinated, supporting a direct role for Dam methylation in replication timing control (Bakker and Smith, 1989). Bakker and Smith further proposed that the transient hemimethylated state generated at daughter origins after replication may itself participate in this timing mechanism (Bakker and Smith, 1989).

This idea was incorporated into the classical model of origin sequestration. Newly replicated *oriC* and the *dnaA* promoter enter a transient sequestered state in which Dam remethylation is delayed, causing these regions to remain hemimethylated for a defined period after replication (Campbell and Kleckner, 1990). Because fully methylated *oriC* is permissive for initiation, this post-replicative hemimethylated interval provides a refractory period that prevents newly replicated origins from immediately reinitiating. Sequestration of the *dnaA* promoter reinforces this control because the promoter contains GATC sites and transient SeqA-dependent hemimethylation represses *dnaA* transcription, thereby reducing DnaA synthesis during the post-initiation period (Campbell and Kleckner, 1990; von Freiesleben et al., 1994).

**Figure 2:**
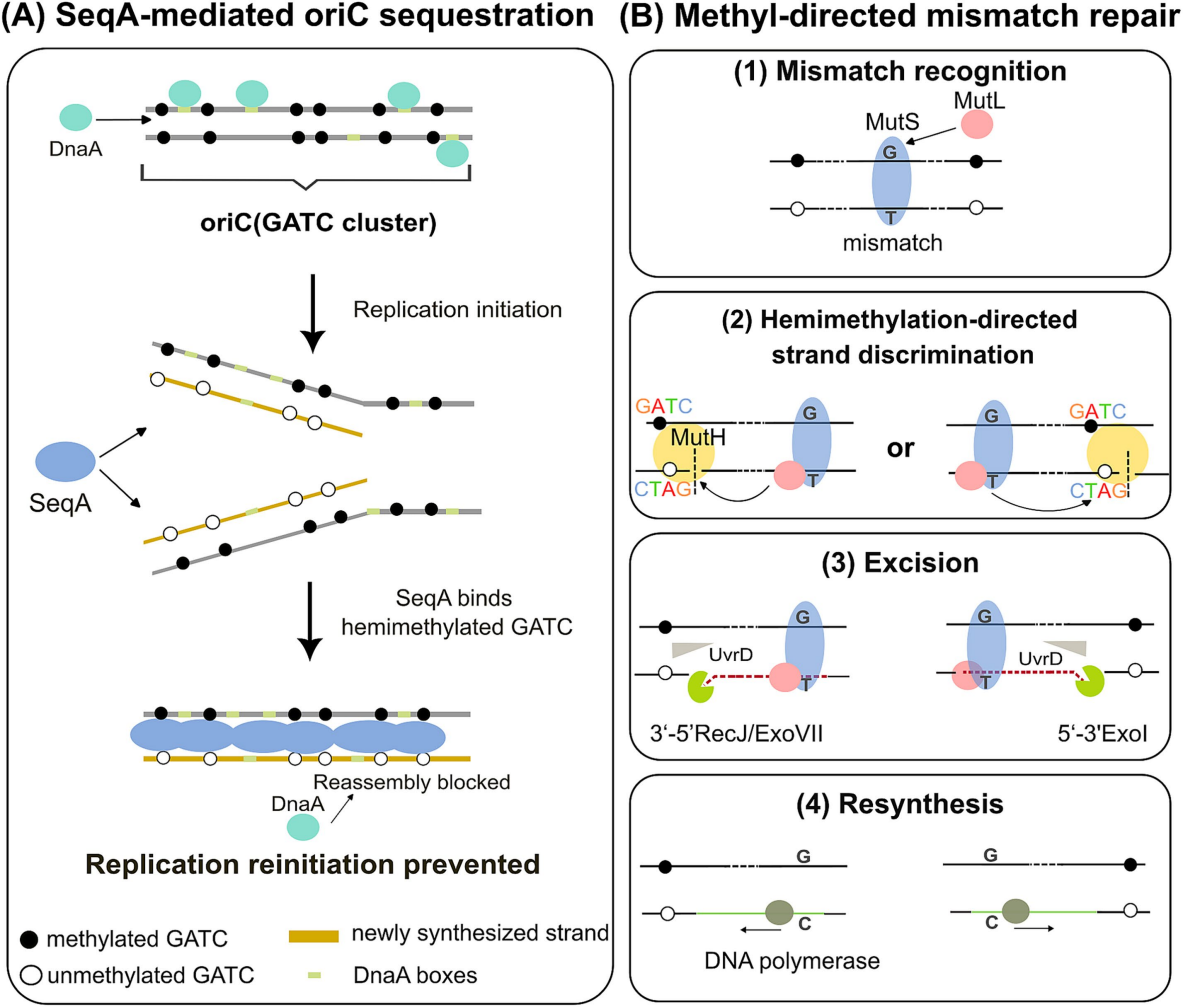
Direct functional readout of hemimethylated DNA in *E. coli*. **(A)** Replication-coupled oriC sequestration. Fully methylated oriC is permissive for DnaA-dependent initiation. After replication, newly replicated origins become hemimethylated at clustered GATC sites. SeqA preferentially binds these hemimethylated clusters, transiently sequestering oriC and preventing immediate reinitiation until Dam-mediated remethylation restores the fully methylated state. **(B)** Hemimethylation-directed strand discrimination during mismatch repair in *E. coli*. MutS recognizes the replication-generated mismatch, while MutL acts as a mediator that couples mismatch recognition to activation of the latent MutH endonuclease. Activated MutH incises the unmethylated daughter strand at a nearby hemimethylated GATC site, providing the strand-discrimination signal for repair. Depending on the position of the nick relative to the mismatch, the error-containing daughter-strand segment is removed by UvrD helicase together with 3′–5′ or 5′–3′ exonuclease activity, followed by DNA resynthesis and ligation.

SeqA provides the molecular link between hemimethylation and origin sequestration. *seqA* mutants lose normal sequestration and display replication overinitiation, identifying SeqA as a major negative regulator of replication initiation (Lu et al., 1994). Biochemical analyzes further showed that SeqA preferentially binds hemimethylated *oriC* DNA, whereas binding to fully methylated or unmethylated substrates is weaker (Slater et al., 1995). By selectively occupying hemimethylated *oriC*, SeqA helps delay Dam-mediated remethylation and maintains the newly replicated origin in a transiently sequestered, initiation-refractory state, thereby preventing immediate reinitiation (Figure 2A). However, although SeqA-dependent sequestration explains why clustered hemimethylated GATC regions such as oriC resist rapid remethylation, how this protected state is released remains incompletely defined. It is unclear whether Dam remethylation resumes from particular GATC sites within the cluster or instead follows broader changes in SeqA occupancy and local origin architecture.

Mechanistic refinements suggest that SeqA does not simply occlude the entire origin. Instead, SeqA preferentially interferes with DnaA binding at low-affinity *oriC* sites, thereby preventing reassembly of an initiation-competent complex without necessarily displacing DnaA from high-affinity sites (Skarstad et al., 2000). In this view, sequestration acts as an origin reset mechanism: hemimethylated *oriC* is maintained temporarily in an initiation-incompetent configuration until Dam remethylation restores the fully methylated, initiation-permissive state. More recent work has broadened the replication-control role of Dam beyond classical *oriC* sequestration. Raghunathan et al. showed that Dam suppresses aberrant *oriC*-independent chromosomal replication, also termed constitutive stable DNA replication (cSDR), and that *dam*-deficient cells can support DnaA-independent viability under genetic conditions that facilitate reverse fork progression (Raghunathan et al., 2019). This finding does not alter the core SeqA–*oriC* model, but it indicates that Dam methylation also contributes to preventing pathological replication restart outside the normal *oriC*-dependent initiation pathway.

### 4.2 Hemimethylation-directed strand discrimination in mismatch repair

Whereas *oriC* sequestration uses hemimethylation to prevent immediate reinitiation, methyl-directed mismatch repair uses the same replication-generated asymmetry to identify the newly synthesized strand. DNA replication generates base mismatches and small insertion–deletion mismatches, and *E. coli* relies on mismatch repair (MMR) to limit replication-associated mutagenesis (Glickman and Radman, 1980; Marinus and Løbner-Olesen, 2014). Genetic evidence connecting Dam methylation to mismatch repair was established by Glickman and Radman, who showed that *mutH*, *mutL*, and *mutS* mutants shared a mutational spectrum with *dam* mutants, including increased base substitutions and frameshift mutations. Their analysis of double and triple mutants further indicated that these genes act in the same methylation-instructed mismatch-correction pathway (Glickman and Radman, 1980). These findings provided early genetic evidence linking Dam methylation to mismatch correction.

A defining problem for MMR is not only detecting a mismatch, but identifying which strand contains the replication error. Using an *E. coli* cell-free repair assay, Lu, Clark, and Modrich showed that repair of heteroduplex DNA is directed by the methylation state of d (GATC) sequences (Lu et al., 1983). In this system, repair synthesis occurred on the unmethylated strand and required ATP together with the *mutH*, *mutL*, *mutS*, and *uvrE* gene products (Lu et al., 1983). These experiments established the basic logic of methyl-directed mismatch repair: after replication, the parental strand remains methylated, whereas the newly synthesized strand is transiently unmethylated, allowing hemimethylated GATC sites to mark the daughter strand for repair. Biochemical studies then separated two steps in this process: Lahue et al. showed that d (GATC) sequences, and even a single hemimethylated GATC site, can direct strand choice, whereas Welsh et al. identified MutH as the methylation-sensitive endonuclease that incises the unmethylated strand at hemimethylated d (GATC) sites (Au et al., 1992; Welsh et al., 1987). Thus, hemimethylation is not simply correlated with strand discrimination; it supplies the strand-specific signal that directs repair initiation.

The pathway was later reconstituted in a purified system containing MutH, MutL, MutS, DNA helicase II/UvrD, SSB, DNA polymerase III holoenzyme, exonuclease I, DNA ligase, ATP, and dNTPs, demonstrating that these factors are sufficient for methyl-directed repair under defined conditions (Grilley et al., 1993; Lahue et al., 1989) (Figure 2B). MutS/MutL-dependent activation of MutH explained how mismatch recognition is coupled to strand-specific incision, and later work showed that excision can proceed from either side of the mismatch depending on the position of the hemimethylated GATC signal (Au et al., 1992; Cooper et al., 1993; Grilley et al., 1993). A nick 5′ to the mismatch supports 5′ → 3′ excision, primarily mediated by RecJ or Exonuclease VII, whereas a nick 3′ to the mismatch supports 3′ → 5′ excision, mainly mediated by Exonuclease I, with additional contributions from Exonuclease VII or Exonuclease X depending on substrate context (Cooper et al., 1993; Grilley et al., 1993). UvrD-dependent unwinding, DNA polymerase III-mediated gap filling, and ligation then restore the repaired duplex. Thus, although the enzymatic route differs according to incision polarity, strand choice remains specified by the unmethylated daughter strand at a hemimethylated GATC site (Cooper et al., 1993; Grilley et al., 1993). More recent work suggests that methyl-directed repair may also be spatially coupled to replication fork movement, with preferential use of hemimethylated GATC sites positioned in the direction of fork progression (Hasan and Leach, 2015). This refinement does not change the core logic of the pathway, but places strand discrimination within the spatial context of ongoing chromosome replication.

### 4.3 Hemimethylation as a competition window in Dam-dependent phase variation

Unlike *oriC* sequestration and mismatch repair, where hemimethylation is read more directly as a timing or strand-discrimination signal, methylation-dependent phase variation uses replication-generated hemimethylation more indirectly, as a transient window in which Dam remethylation and regulatory protein rebinding compete. Unlike phase variation mechanisms driven by DNA rearrangement or slipped-strand mispairing, Dam dependent systems switch between transcriptionally ON and OFF states without changing the underlying DNA sequence. Early work on the *pap* operon showed that phase switching is associated with differential methylation of GATC sites in the regulatory region rather than DNA rearrangement itself (Blyn et al., 1990).

In methylation-dependent phase variation, replication transiently disrupts pre-existing methylation patterns and protein–DNA complexes (Casadesus and Low, 2006; Correnti et al., 2002; Kaminska and van der Woude, 2010). The resulting hemimethylated DNA creates a short interval in which Dam remethylation and regulatory protein rebinding compete at overlapping sites. Hemimethylation therefore does not itself encode the ON or OFF state; rather, it provides the post-replicative window in which the previous state is either restored or replaced (Casadesus and Low, 2006).

The *pap* operon of uropathogenic *E. coli* established the classical model for this regulatory logic. In this system, alternative transcriptional states are associated with differential Lrp occupancy and reciprocal methylation patterns at regulatory GATC sites. In OFF cells, Lrp binding protects specific GATC sites from Dam methylation and maintains transcriptional repression; in ON cells, PapI and Dam-dependent methylation promote an alternative Lrp occupancy pattern that supports transcription (Blyn et al., 1990; Hernday et al., 2003). Replication-generated hemimethylated intermediates provide the transition point at which Dam, Lrp, and PapI can re-compete for the regulatory region after fork passage (Hernday et al., 2003). Biochemical analysis of the *pap* regulatory region further supports this competition model by showing that Lrp and Dam assembly is mutually influenced at regulatory GATC-containing sites (Peterson and Reich, 2008). Thus, *pap* phase variation is best understood not as a simple methylation-state conversion, but as replication-coupled reorganization of methylation and regulatory protein occupancy (Hernday et al., 2003).

**Figure 3:**
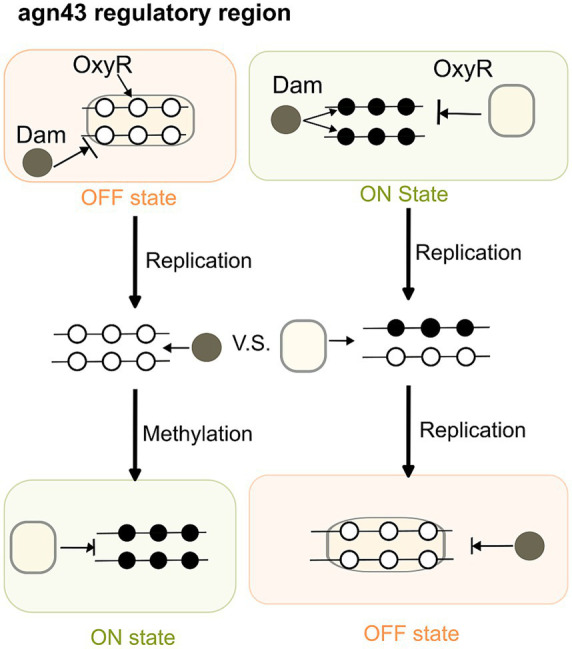
Replication-coupled hemimethylation creates a switching window for Dam–OxyR-dependent phase variation at agn43. The agn43 promoter region contains three closely spaced GATC sites that overlap with the OxyR-binding region. In the OFF state, OxyR binding protects these GATC sites from Dam methylation and represses agn43 transcription. Following DNA replication, transient strand-asymmetric methylation states create a competition window in which either Dam-mediated remethylation or OxyR rebinding can predominate. Dam methylation prevents OxyR binding and permits transcription, thereby favoring the ON state, whereas OxyR rebinding protects the region from methylation and maintains or restores the OFF state. This competition enables reversible switching between alternative transcriptional states across replication cycles.

In the *E. coli* K-12 lineage, the best-characterized example is Ag43 phase variation. Expression of *agn43* is controlled by competition between Dam-dependent methylation and OxyR-mediated repression (Haagmans and van der Woude, 2000; Henderson and Owen, 1999). The *agn43* regulatory region contains three GATC sites whose methylation state correlates with transcriptional state. In the OFF phase, OxyR binding protects these sites from Dam methylation and represses transcription; methylation of the same sites inhibits OxyR binding and relieves repression (Haagmans and van der Woude, 2000; Waldron et al., 2002). This arrangement places Dam and OxyR in direct competition at overlapping regulatory DNA elements (Figure 3).

Replication links this competition to hemimethylation. Newly replicated *agn43* DNA passes through a hemimethylated intermediate in which Dam and OxyR can re-compete for the regulatory region. Replication-associated competition is supported by the altered Ag43 switching bias observed in seqA mutants, whereas later work indicates that OxyR-mediated, rather than SeqA-mediated, sequestration of hemimethylated agn43 DNA is central to establishing and maintaining the OFF state (Correnti et al., 2002; Kaminska and van der Woude, 2010). Within this window, if Dam remethylates the region before OxyR binding is restored, the methylated, transcriptionally permissive state is maintained. If OxyR rebinds before remethylation is completed, methylation is blocked and the OFF state is re-established. In this model, hemimethylation acts as a transient post-replicative intermediate that exposes the regulatory region to renewed competition between Dam and OxyR, rather than as a stable transcriptional mark (Correnti et al., 2002).

Together, *pap* and *agn43* show that hemimethylation can support epigenetic transcriptional switching through a mechanism distinct from both *oriC* sequestration and mismatch repair. In *oriC* control, hemimethylation contributes to a refractory period that prevents immediate reinitiation. In MMR, it marks the newly synthesized strand for repair. In phase variation, it creates a post-replicative competition window in which methylation and regulatory protein occupancy are reassembled into alternative transcriptional states.

These classical mechanisms were established largely through indirect evidence, including mutant-based functional assays, restriction enzyme analysis, remethylation kinetics, footprinting, and reporter systems. Such approaches defined the functional consequences of hemimethylation with considerable strength, but they could not directly visualize strand-paired hemimethylated configurations on individual newly replicated DNA duplexes. This gap provides the rationale for considering how genome-wide, single-molecule, and duplex-aware sequencing technologies can now revisit replication-coupled hemimethylation at higher molecular resolution.

## 5 Why classical assays inferred rather than directly observed hemimethylation

Global assays answer a limited first question: how much modified base is present in bulk DNA. Global quantitative approaches, such as HPLC, LC–MS/MS, and antibody-based dot blot assays, measure modified nucleotides or modification-specific signals in bulk genomic DNA. HPLC and LC–MS/MS quantify modified nucleosides after DNA hydrolysis, whereas antibody-based dot blots provide an immunochemical readout of overall modification abundance (Armstrong et al., 2011; Boulias and Greer, 2021; Jia et al., 2017). These assays can detect the presence and overall level of DNA modifications, including 5mC and, in appropriate LC–MS/MS workflows, bacterial modifications such as 6mA and 4mC, but they do not provide genomic position, strand identity, or the paired methylation configuration of both strands within the same DNA duplex.

Locus-specific assays increased positional resolution but remained population averaged. Among these approaches, restriction enzyme–based assays provided one of the most widely used strategies for interrogating methylation at known motifs or selected loci. Restriction enzyme based methylation assays infer methylation status by exploiting the fact that site-specific DNA methylation can inhibit, reduce, or alter cleavage by restriction endonucleases in an enzyme- and motif-specific manner (Nelson et al., 1993). In locus-specific studies, these restriction-based readouts were often combined with Southern blotting, footprinting, or reporter assays to connect methylation state with local protein binding or transcriptional output. These approaches were central to classical studies of *oriC* sequestration, methyl-directed mismatch repair, and *agn43*/*pap* phase variation (Campbell and Kleckner, 1990; Haagmans and van der Woude, 2000; Lahue et al., 1987). However, the cleavage response to methylated DNA, including hemimethylated substrates, is highly enzyme-specific and often must be determined empirically (Nelson et al., 1993). Thus, restriction-based approaches are informative when the relevant motif and enzyme specificity are known, but they are constrained by the available repertoire of methylation-sensitive or methylation-dependent enzymes and are less suited to unbiased discovery of novel bacterial methylation motifs (Nelson et al., 1993). More importantly for hemimethylation, a restriction pattern, footprint, or reporter signal represents an ensemble of DNA molecules. Such assays could support strong functional inference about hemimethylation, but they could not directly determine the methylation configuration of an individual DNA molecule.

Genome-wide sequencing methods expanded methylation analysis from selected loci to base-resolution maps across the genome. A representative example is bisulfite sequencing, which has long been regarded as a gold-standard approach for mapping eukaryotic 5mC (Beaulaurier et al., 2019). In this approach, bisulfite treatment converts unmethylated cytosines to uracil, which is read as thymine after sequencing, whereas 5mC is relatively resistant to conversion and is therefore retained as cytosine in the sequencing readout. This chemistry allows cytosine methylation to be mapped at single-base resolution and has been used to characterize Dcm-dependent 5mC methylation and associated stationary-phase transcriptional effects in *E. coli* K-12 (Kahramanoglou et al., 2012). However, bisulfite-based strategies remain chemically constrained. Conventional bisulfite sequencing primarily captures cytosine methylation and does not detect Dam-dependent 6mA (Beaulaurier et al., 2019). TET-assisted bisulfite approaches can extend cytosine-based mapping to some 4mC positions, but 6mA remains undetectable, limiting the utility of bisulfite-based methods for bacterial methylomes in which adenine methylation is prevalent (Yu et al., 2015). More importantly for hemimethylation, genome-wide positional resolution does not by itself preserve strand-paired dyad information. Even when sequencing-based methods report methylation fractions at individual sites, these fractions remain population-level summaries: a site reported as 50% methylated could reflect a mixture of fully methylated and unmethylated molecules, a population enriched for hemimethylated molecules, or other combinations of strand states. These methodological distinctions are summarized in Table 2.

**Table 2:** Resolution hierarchy of bacterial methylation detection methods.

Detection level	Representative methods	Primary observable output	Hemimethylation resolution
Global bulk	HPLC/LC–MS/dot blot	Global modification abundance	No hemimethylation resolution
Locus-specific	Restriction digest/Southern blot/reporter assays	Methylation state at specific loci	Indirect inference at selected loci
Genome-wide population	Bisulfite sequencing	Site-level methylation fraction	Population-level inference from site fractions
Single-molecule long-read	SMRT sequencing	Per-read polymerase-kinetic modification signatures	Single-molecule inference from kinetic signals
Single-molecule long-read	Simplex nanopore sequencing	Per-read ionic-current modification signatures	Single-molecule inference from current signals
Duplex-resolved	Duplex-aware nanopore sequencing	Strand-paired duplex methylation configurations	Direct duplex-level hemimethylation resolution

## 6 What single-molecule and duplex-aware sequencing now enables

Single-molecule long-read sequencing changed bacterial methylome analysis by allowing DNA modifications to be detected from native DNA molecules without bisulfite conversion or restriction digestion (Flusberg et al., 2010). SMRT sequencing provides a key example of this transition. In this platform, nucleotide sequence is read from fluorescently labeled dNTP incorporation during real-time DNA synthesis, whereas DNA modifications are inferred from perturbations in polymerase kinetics, particularly changes in inter-pulse duration (IPD), the time interval between successive incorporation pulses (Eid et al., 2009; Flusberg et al., 2010; Schadt et al., 2013). Covalent DNA modifications can alter these kinetic signatures, enabling detection of the major bacterial methylation types, including 6mA, 4mC, and 5mC, although with different sensitivities because each modification produces a distinct signal-to-noise profile (Beaulaurier et al., 2015; Blow et al., 2016; Murray et al., 2012; Schadt et al., 2013). In bacterial genomes, this capability enabled genome-wide methylation motif discovery and helped establish that bacterial methylation is often organized around highly specific sequence motifs (Beaulaurier et al., 2019; Blow et al., 2016). The strongest native SMRT signals are generally observed for 6mA and 4mC, whereas 5mC and 5hmC produce weaker kinetic signatures and may require higher sequencing coverage or additional conversion-based strategies to improve detection (Clark et al., 2013; Schadt et al., 2013). However, although SMRT reads can distinguish strand orientation during sequencing, standard SMRT workflows generally do not preserve complementary strands from the same original DNA duplex as linked strand-paired information. As a result, hemimethylation usually remains inferred rather than directly resolved at the duplex level.

Nanopore sequencing provides a parallel native-DNA approach in which DNA molecules are analyzed through ionic current signals generated as single-stranded DNA passes through a protein nanopore (Clarke et al., 2009; Jain et al., 2016; Laszlo et al., 2014). Because the measured current reflects the short k-mer occupying the pore rather than a single isolated base, both canonical sequence context and covalent base modifications can influence the signal (Laszlo et al., 2014; Rand et al., 2017; Simpson et al., 2017). Early work showed that nanopore current signals could distinguish 5mC from unmodified cytosine, establishing the feasibility of modification detection from native DNA without chemical conversion (Laszlo et al., 2013; Simpson et al., 2017). Early nanopore approaches included supervised or comparison-based models that often required training data, known contexts, or methylation-free controls (Rand et al., 2017; Stoiber et al., 2017). In parallel, nanoraw explored genome-guided *de novo* signal-level discovery without prior training data, but remained an early preprint-stage framework (Stoiber et al., 2017). Later tools broadened this framework. Nanodisco extended nanopore methylation discovery to 6mA, 4mC, and 5mC across bacterial and microbiome samples, addressing the need to identify diverse bacterial methylation types and motifs without restricting analysis to eukaryotic 5mC contexts (Tourancheau et al., 2021).

Nevertheless, nanopore methylation analysis remains sensitive to sequence context, modification type, basecalling models, and sequencing chemistry. Modification-associated signal shifts can complicate basecalling and produce systematic errors in bacterial reads (Chiou et al., 2023). Benchmarking studies indicate that newer nanopore chemistries and basecalling models have reduced some systematic errors, but false-positive and false-negative methylation calls remain context dependent (Ni et al., 2023). These limitations have motivated continued method development to improve the robustness of nanopore-based bacterial methylation detection. For example, rather than relying only on raw-current inference, Hammerhead uses modification-induced, strand-specific mismatch patterns as an alternative signal for de novo bacterial DNA modification discovery (Liu et al., 2024). These nanopore simplex approaches improve molecule-level phasing but still do not by themselves distinguish whether partial methylation reflects true hemimethylated dyads.

Compared with bulk and locus-specific assays, genome-wide sequencing methods improved positional resolution by assigning methylation signals to individual genomic sites. SMRT and nanopore sequencing move one level further by preserving methylation information along individual long DNA molecules. This allows methylation states at multiple motifs to be phased along the same read, reveals molecule-to-molecule heterogeneity, and provides long-range genomic context that is unavailable to bulk assays or most short-read methods. For hemimethylation, this richer single-molecule information can support more constrained inference than population-level methylation fractions alone, especially when partial methylation, strand-biased signals, or molecule-level methylation patterns are observed. Nevertheless, single-molecule resolution is not equivalent to duplex resolution. Standard SMRT and simplex nanopore reads generally do not link the methylation states of both complementary strands from the same original DNA duplex. Therefore, they narrow the interpretive gap between population-level methylation fractions and molecular methylation patterns, but they still do not directly resolve the defining feature of hemimethylation: asymmetric methylation across paired strands of the same DNA molecule.

**Figure 4:**
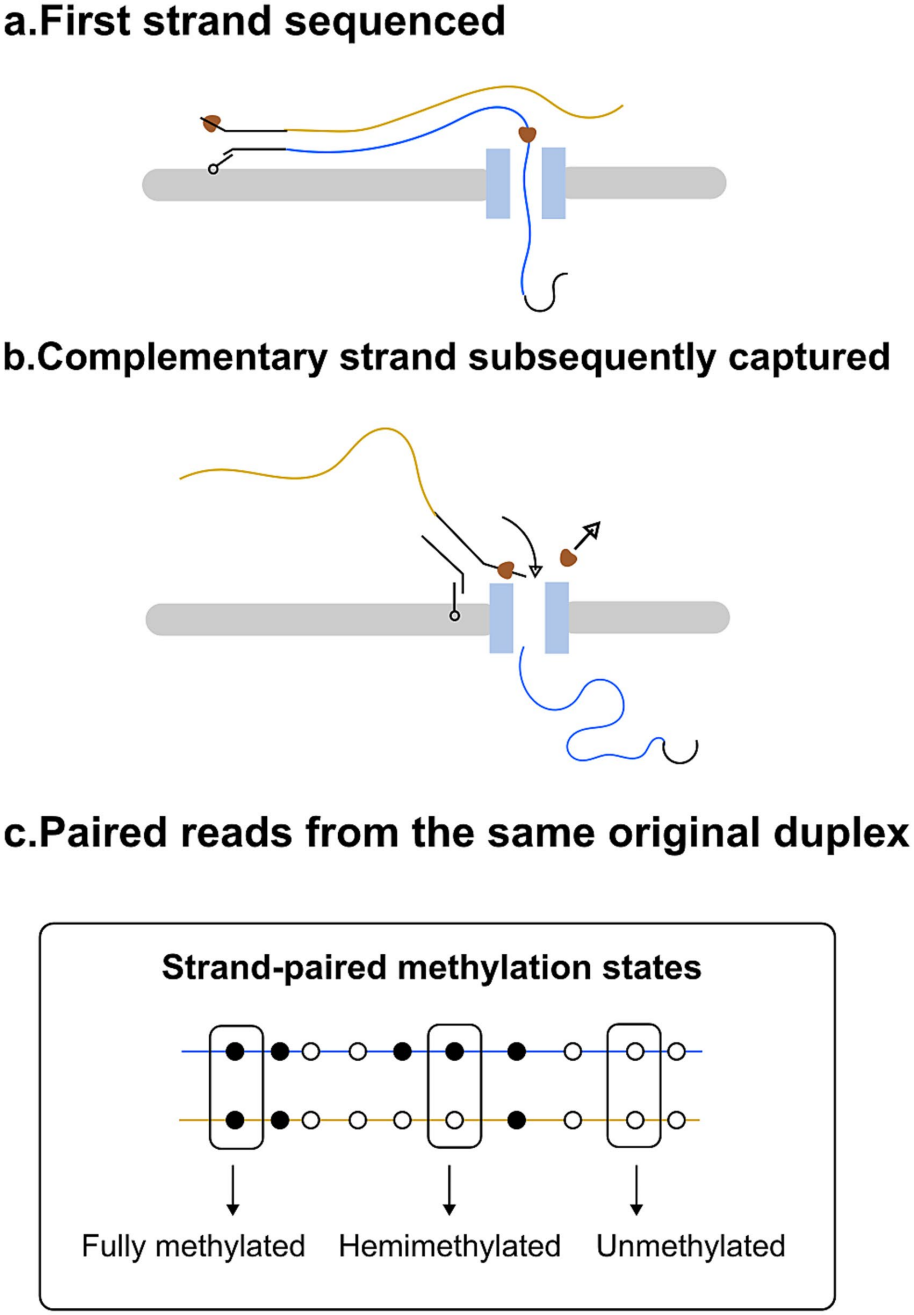
Duplex-aware nanopore sequencing preserves strand-paired methylation information. In duplex-aware nanopore sequencing, one strand of an adapted double-stranded DNA molecule is first sequenced through the nanopore. In a subset of molecules, the complementary strand is subsequently captured and sequenced, allowing the two reads to be paired as originating from the same original DNA duplex. This paired-strand context enables methylation calls to be compared across complementary strands, thereby allowing fully methylated, hemimethylated, and unmethylated dyad states to be distinguished conceptually. Filled circles indicate methylated sites, open circles indicate unmethylated sites, and blue and gold lines indicate complementary DNA strands from the same original duplex. The brown oval represents the motor protein associated with the sequencing adapter, which controls strand translocation through the nanopore; the small open circle represents an adapter/tether element that helps retain the complementary strand near the pore and facilitates subsequent capture. Duplex reads represent a subset of total nanopore reads; in an ONT-derived instructional summary of R10.4.1 flowcells with v14 chemistry, duplex reads were described as accounting for fewer than 50% of reads, whereas reads lacking paired complementary-strand information remain simplex (Black, 2023). The duplex sequencing workflow was redrawn and simplified based on publicly available instructional material licensed under CC-BY 4.0, with modifications.

Duplex-aware nanopore sequencing addresses the specific limitation that standard simplex reads do not preserve the paired methylation states of both strands from the same original DNA duplex. In ONT duplex basecalling, complementary strands that pass through the pore in succession can be associated, allowing modified-base calls to be interpreted in a paired-strand context (ONT, 2025a) (Figure 4). Recent work benchmarking nanopore methylation detection in mouse cerebellum whole-genome DNA has shown that strand-asymmetric 5mC and 5hmC states can be studied at genome scale, demonstrating the value of duplex-paired reads for resolving cytosine modification configurations (Halliwell et al., 2025). Current ONT analysis tools also explicitly support hemi-methylation outputs from duplex modified-base calls, with Modkit aggregating paired modification calls at motif positions for each double-stranded molecule (ONT, 2025b). Conceptually, this is the first class of approaches that directly matches the definition of hemimethylation as an asymmetric modification state across paired strands of the same DNA duplex.

Similar strand-paired logic has also been implemented outside nanopore sequencing. Methyl-CODEC is an NGS-based duplex methylation method that combines enzymatic cytosine-methylation conversion with preservation of complementary-strand information from the same original DNA molecule. Using this design, Liu et al. showed that methylation states can be interpreted in a paired-duplex context, allowing hemimethylation to be distinguished from unlinked site-level methylation fractions (Liu et al., 2025). Although Methyl-CODEC was developed outside bacterial methylome analysis, it reinforces the central methodological point that hemimethylation is a paired-duplex property and can only be resolved directly when modification information from both strands of the same original DNA molecule is retained (Liu et al., 2025).

However, several technical constraints remain for duplex nanopore sequencing. Duplex-aware methylation analysis requires both complementary strands of the same original DNA molecule to be captured and paired, which substantially reduces the effective read depth available for dyad-level analysis. In a recent duplex nanopore study of cytosine modifications, duplex-paired reads accounted for a mean of 32% of sequence reads, and 29–33 × median genomic coverage yielded only 2–2.5 × mean dyad depth; the authors therefore recommended total coverage at least 6–7 times higher than the desired duplex depth when duplex capture is the objective (Halliwell et al., 2025). Moreover, although duplex nanopore sequencing has demonstrated strand-asymmetric 5mC/5hmC analysis (Halliwell et al., 2025), broader routine detection of modifications such as 6mA remains dependent on further improvements in basecalling models and validation.

## 7 Discussion

The classical Dam-dependent systems of *E. coli* K-12 show that bacterial DNA methylation should not be understood only as a stable, motif-specific genomic mark. After DNA replication, fully methylated GATC sites transiently become hemimethylated, creating a short-lived strand-asymmetric state that can be read by cellular machineries. In *oriC* sequestration, this state contributes to a refractory period that prevents immediate reinitiation; in methyl-directed mismatch repair, it identifies the newly synthesized strand; and in Dam-dependent phase variation, it creates a post-replicative competition window in which methylation and regulatory protein binding are re-established or switched (Campbell and Kleckner, 1990; Haagmans and van der Woude, 2000; Lu et al., 1983).

These mechanisms explain why bacterial hemimethylation deserves renewed attention. Classical studies established its biological importance through genetic, biochemical, restriction-based, footprinting, and reporter assays, but they generally inferred hemimethylated states from functional outcomes rather than directly observing paired strands within individual DNA molecules. As a result, several mechanistic questions remain unresolved. For example, in *oriC* sequestration, why prolonged SeqA-dependent protection is concentrated at *oriC* and the *dnaA* promoter, and how sequestration is terminated, remain incompletely understood (Slater et al., 1995).

A further gap concerns methylation systems beyond Dam. Dcm-dependent 5mC methylation in *E. coli* K-12 has been linked to very-short-patch repair and growth phase-associated transcriptional effects, but classical mechanisms explicitly built around Dcm-generated hemimethylated intermediates have not yet been clearly established (Kahramanoglou et al., 2012; Lieb and Bhagwat, 1996). Whether this reflects a true biological difference between Dam and Dcm systems, or simply the historical difficulty of detecting transient strand-paired states, remains an open question.

Recent single-molecule and duplex-aware sequencing technologies provide a route to revisit these problems. SMRT and nanopore sequencing have expanded bacterial methylome analysis from bulk or locus-specific inference toward genome-wide, molecule-level methylation profiles (Blow et al., 2016; Flusberg et al., 2010; Tourancheau et al., 2021). Duplex-aware approaches go one step further by preserving paired-strand information, making it possible in principle to distinguish fully methylated, hemimethylated, and unmethylated dyads within individual DNA molecules (Halliwell et al., 2025; Liu et al., 2025). Although current applications remain limited by duplex depth, modification-calling accuracy, and the need for validation at bacterial motifs such as GATC, these technologies shift the central question from whether bacterial motifs are methylated to when, where, and on which strand methylation states arise after replication. This shift may reveal whether replication-coupled hemimethylation is restricted to a few classical Dam-dependent mechanisms or represents a broader, previously under-observed layer of bacterial epigenetic regulation.
